# Two sides of the same coin: a complex presentation of autosomal dominant tubulointerstitial kidney diseases: a literature review and case reports

**DOI:** 10.3389/fped.2023.1283325

**Published:** 2023-11-10

**Authors:** Margareta Fistrek Prlic, Sanda Huljev Frkovic, Bodo Beck, Ivana Tonkovic Durisevic, Stela Bulimbasic, Marijana Coric, Lovro Lamot, Ema Ivandic, Ivana Vukovic Brinar

**Affiliations:** ^1^ Department of Nephrology, Arterial Hypertension, Dialysis and Transplantation, University Hospital Center Zagreb, Zagreb, Croatia; ^2^ Department of Pediatrics, Division of Genetics and Metabolism, University Hospital Center Zagreb, Zagreb, Croatia; ^3^ University of Zagreb, School of Medicine, Zagreb, Croatia; ^4^ Institute of Human Genetics, University of Cologne, Cologne, Germany; ^5^ Department of Laboratory Diagnostics, Division of Cytogenetics, University Hospital Center Zagreb, Zagreb, Croatia; ^6^ Department of Pathology, University Hospital Center Zagreb, Zagreb, Croatia; ^7^ Department of Pediatrics, Division of Nephrology, Dialysis and Transplantation, University Hospital Center Zagreb, Zagreb, Croatia

**Keywords:** hereditary kidney disease, autosomal dominant inheritance, tubulointerstitial kidney disease, kidney cyst, renal failure, case report

## Abstract

**Introduction:**

Genetic kidney diseases are underdiagnosed; namely, from 7% to 40% of patients suffering from chronic kidney disease (CKD) can carry a pathogenic variant, depending on population characteristics. Hereditary tubulointerstitial kidney diseases, including autosomal dominant tubulointerstitial kidney diseases (ADTKD), are even more challenging to diagnose. ADTKD is a rare form of genetic kidney disease resulting from pathogenic variants in the *MUC1, UMOD, HNF1B, REN, SEC61A1,* and *DNAJB11* genes. There is no typical clinical or histopathological sign of ADTKD, it is characterized by progressive CKD, an autosomal dominant inheritance pattern, and tubular atrophy with interstitial fibrosis on kidney biopsy. There is no significant proteinuria, and the urinary sediment is bland. The patients usually do not have severe arterial hypertension. There can be a history of early gout, especially when compared to the UMOD gene variants. Children can have enuresis due to a loss of renal concentration. On ultrasound, the kidneys can appear normal or small in size. Renal cysts are not pathognomonic for any of the named diseases. End-stage renal disease (ESRD) develops at the average age of 45, but this can be very variable. Family history that suggests autosomal dominant inheritance and CKD fulfilling the aforementioned characteristics of tubulointerstitial kidney disease should raise suspicion of ADTKD. In the setting of a negative family history for CKD, clinical suspicion should be raised based on clinical characteristics, including early onset of hyperuricemia or gout and compatible histology on the kidney biopsy. Contrary to the aforementioned characteristics of ADTKD, in the case of HNF1B-related disease, there is a more complex clinical presentation with extrarenal manifestations of the disease (diabetes mellitus, hypomagnesemia, neurologic and psychiatric disturbances, etc.). The diagnosis of ADTKD is based on a positive family history and a detection of the pathogenic variant in one of the genes in an affected individual.

**Aim:**

The aim of our study is to present two case reports of ADTKD with different characteristics (slowly progressive CKD vs. complex clinical presentation with an extrarenal manifestation of the disease) with a literature review.

**Methods:**

A 34-year-old patient with CKD and a positive family history of CKD in whom kidney biopsy showed nonspecific chronic changes, with only genetic analysis confirming the diagnosis of MUC1-related ADTKD. Our second case is of a 17-year-old patient with an unremarkable family history who was initially referred to genetic counseling due to cognitive and motor impairment with long-lasting epilepsy. Extensive workup revealed increased serum creatinine levels with no proteinuria and bland urinary sediment, along with hypomagnesemia. His genetic analysis revealed 17q12 deletion syndrome, causing the loss of one copy of the *HNF1B* gene, the *AATF,* and the *LHX1* gene.

**Conclusion:**

Autosomal dominant tubulointerstitial kidney diseases are challenging to diagnose due to a lack of typical clinical or histopathological signs as well as an uncharacteristic and versatile clinical presentation. Increased clinical awareness is crucial for the detection of these diseases.

## 1. Introduction

It is unknown how common inherited kidney diseases are, but they are certainly more frequent than diagnosed. There are more than 200 hereditary kidney diseases described worldwide (1, 2). A systematic approach using exome sequencing in more than 3,000 patients with CKD in Germany found diagnostic variants in almost 10% of patients, but this can vary from 7 to 40% depending on population characteristics (3, 4). Among the hereditary tubulointerstitial kidney diseases, the autosomal dominant tubulointerstitial kidney diseases (ADTKD) resulting from mutations in the *MUC1, UMOD, HNF1B, REN, SEC61A1*, and *DNAJB11* genes, are among the commonest. ADTKD-*UMOD* alone is estimated to be responsible for 3% of the genetic causes of kidney disease worldwide, being the third most common genetic cause of nephropathy after autosomal dominant polycystic kidney disease and Alport syndrome (3). Moreover, ADTKD-*MUC1* is the second most prevalent form of ADTKD, although the overall prevalence of ADTKD could be higher considering challenges in identifying pathogenic variants of *MUC1* through non-NGS genetic testing (5). While ADTKD is not a common genetic cause of renal impairment, the versatile clinical presentation, unavailability of genetic testing, and relative lack of awareness make it hard to diagnose in everyday clinical practice (6, 7). The aim of our manuscript is therefore to present two case reports of ADTKD with different characteristics (slowly progressive CKD vs. complex clinical presentation with an extrarenal manifestation of the disease) along with a narrative literature review.

### 1.1. Presentation, classification, and diagnosis of ADTKD

There is no typical clinical or histopathological sign of ADTKD. The whole group is characterized by progressive CKD, the findings of tubular atrophy and interstitial fibrosis in kidney biopsy, and an autosomal dominant inheritance pattern (5–10). Usually, there is no significant proteinuria, and the urinary sediment is bland (5, 6). The patients typically do not have severe arterial hypertension early in the course of the disease. There can be a history of early gout, especially with the *UMOD* gene variants (6). Children have enuresis due to a loss of renal concentration ability.6 ESRD develops by the age of 45, but this can vary among cases (11–13). On ultrasound, the kidneys appear normal or small in size (6, 13). Disease related to variants *MUC1, UMOD, SEC61A1*, and *REN* is related to the kidney, whereas disease related to HNF1B has a more complex clinical presentation related to many organs (13). The disease related to *DNAJB11* can mimic autosomal dominant polycystic kidney disease (ADPKD). 6 Renal cysts are not pathognomonic for any of the named diseases. Histological changes in the kidney are also not typical—tubular atrophy and fibrosis, lamellation, and thickening of tubular basement membranes, and tubular microcysts can be found. Immunofluorescence for complement and immunoglobulins is negative (13). Multiple names have been used for ADTKD in the past, including ‘Medullary Cystic Kidney Disease type 1’ for the disease caused by *MUC1* mutations, and ‘Medullary Cystic Kidney Disease type 2’, ‘Familial Juvenile Hyperuricemic Nephropathy’ for ADTKD-*REN*, ‘Uromodulin-Associated Kidney Disease’ for *UMOD* related diseases, which created confusion (13). The gene-based classification, genetic diagnosis, as well as clinical presentation of different types of ADTKD is presented in Table 1. Each one of the associated genes encodes a protein with a specific function related to the normal performance of the kidney and other organs. In short, the *MUC1* gene produces mucin-1, the *UMOD* gene encodes uromodulin, also known as the Tamm-Horsfall protein, the *HNF1B* gene produces hepatocyte nuclear factor 1 beta, the *SEC61A1* gene produces translocon subunit *SEC61A*, and the *DNAJB11* gene produces the cofactor of GRP78/BiP (6–10).

**Table 1:** The gene-based classification, genetic diagnosis, and clinical presentation of different types of ADTKD.

Causal gene	OMIM#	Chromosome/exons	Proposed terminology	Previously used terminology	Clinical presentation
*UMOD*	191845	16p12.3711	ADTKD–*UMOD*	UKD (uromodulin kidney disease), UAKD uromodulin-associated kidney disease), FJHN (familial juvenile hyperuricemic nephropathy), CKD2 (medullary cystic kidney disease type 2)	CKD, hyperuricemia gout, renal cysts
*MUC1*	158340	1q22/8	ADTKD–*MUC1*	MKD (mucin-1 kidney disease), MCKD1 (medullary cystic kidney disease type 1)	CKD, hyperuricemia, gout, renal cysts
*REN*	179820	1q32.1/10	ADTKD–*REN*	FJHN2 (familial juvenile hyperuricemic nephropathy type 2)	Anemia, hypotension,
					Hyperkalemia, CKD
					Hyperuricemia, gout, renal cysts
*HNF1B*	189907	17q12/9	ADTKD–*HNF1B*	MODY5 (maturity-onset diabetes mellitus of the young type 5), RCAD (renal cyst and diabetes syndrome)	CAKUT, hypomagnesemia
					Neurodevelopmental disorders, diabetes mellitus, CKD
					Hyperuricemia, gout
					Renal cysts, pancreatic and liver dysfunction
*SEC61A1*	617056	3q21.3/12	ADTKD-*SEC61A1*		Growth retardation, anemia, neutropenia, RTIs, CKD
					Hyperuricemia, gout, renal cysts
*DNAJB11*	618061	3q27.3/10	ADTKD- *DNAJB11*		Small renal cysts, liver cysts, enlarged kidneys

ADTKD, autosomal dominant tubulointerstitial kidney disease; CKD, chronic kidney disease; CAKUT, congenital anomalies of the kidney and urinary tract; RTI, respiratory tract infections.

### 1.2. ADTKD-*UMOD*

In ADTKD related to variants in the *UMOD* gene, the accumulation of the disarranged uromodulin protein inside the endoplasmic reticulum of epithelial cells in the renal tubules leads to tubular cell death. This process leads to chronic kidney failure (14). ADTKD-*UMOD* patients have slowly progressive kidney damage, usually in the second or third decade of life, with bland urinary sediment and slightly elevated proteinuria (1, 6, 13). The ability to maintain urinary concentration is impaired, but not clinically relevant. Changes in kidney histology are unspecific, but can mimic focal segmental glomerulosclerosis (FSGS) (14). However, electron microscopy can visualize uromodulin accumulation as intracellular deposits of amorphous or fibrillar material in the endoplasmatic reticulum of Henle's loop (15, 16). Kidney ultrasound may reveal kidney cysts, and kidneys can be small or normal in size. 1 Hyperuricemia is often present, usually precedes CKD, and is more prevalent in men (17). The diagnosis is established by genetic testing, revealing a heterozygous pathogenic variant in the *UMOD* gene. More than 130 pathogenic variants have been described, predominantly missense changes clustering in exons 3 and 4, leading to the misfolding of the uromodulin protein (5, 18, 19). In the ADTKD cohort, the median age of ESRD onset was 54 years, and women seem to have better renal survival than men. 17 No specific treatment is available for ADTKD-*UMOD*. Usual therapy is similar to treatment for other forms of CKD. Hyperuricemia is treated with allopurinol or febuxostat, which can prevent gout attacks. However, there is no data suggesting that therapy with this agent could slow down the progression of CKD (1, 13). Interestingly, disease progression was slowed in an animal model of ADTKD-*UMOD* after treatment with a tumor necrosis factor-α inhibitor, emphasizing a potential role of inflammation in ADTKD-*UMOD* development (20).

### 1.3. ADTKD-*MUC1*

The *MUC1* gene is located on chromosome 1q21, and it encodes mucin 1, a transmembrane protein, expressed on the apical surface of many cells. Mucin 1 provides a protective cellular lair but is also involved in cellular signaling (18). In the kidney, mucin 1 is expressed in the distal tubule and collecting duct (18). Most *MUC1* mutations are caused by cytosine duplication within a seven-cytosine stretch in the variable-number tandem repeat (VNTR) region, that usually produces a frameshift mutation (21). Other mutations include the addition of a guanosine residue or the loss of two cytosine residues. The mutated gene produces an abnormal protein, and retention of the changed protein in the renal tubular epithelial cells leads to kidney damage. A positive staining of abnormal proteins in the urinary exfoliated cells could help diagnose the condition (21). The true prevalence of ADTKD-*MUC1* is unknown, as pathogenic variants in *MUC-1* are not detected by NGS analysis. The diagnostic procedure requires specialized diagnostic testing, which is not routinely performed. The main clinical features of ADTKD-*MUC1* are the same as in ADTKD-*UMOD*, i.e., chronic kidney disease, hyperuricemia, gout, bland urinary sediment, and dominant inheritance. The renal disease appears to be more aggressive in patients with ADTKD-*MUC1* in comparison to ADTKD-*UMOD* with an earlier onset of ESDR (mean age 45 years) (17). There is no specific therapy for ADTKD-*MUC1*. However, the newly identified molecule BRD4780 induces the removal of changed proteins and could be a promising solution for toxic proteinopathies, such as ADTKD-*MUC1* or ADTKD-*UMOD* (22).

### 1.4. ADTKD-*REN*

The autosomal dominant tubulointerstitial kidney disease related to *REN* variants was previously known as Familial Juvenile Hyperuricemic Nephropathy Type 2. The *REN* gene is located on chromosome 1, and the disease is mostly caused by mutations in the first exon of the REN gene. The type of mutation is either a missense change or an in-frame deletion. These mutations lead to improper renal development (23). Renin has a vital role in the renin-angiotensin-aldosterone system, which has a crucial role in a lot of biological functions, e.g., sodium and potassium balance, vascular tone modification, etc (24). ADTKD-*REN* has an early onset during childhood, and rarely presents as a milder form of the disease in adulthood.6 Similar to other forms of ATDKD, ADTKD-*REN* doesn't have any specific clinical or pathohistological features, except for decreased immunohistochemical staining for renin in the juxtaglomerular apparatus (18). However, due to decreased secretion of renin and aldosterone, hypotension and hyperkalemia can be observed (21, 25). Patients suffering from ADTKD—*REN* are susceptible to acute renal injury in setting of hypovolemia and NSAID use (18, 24). Despite early onset and reduced GFR in childhood, kidney function remains stable during adolescence (26). Another sign of ADTKD-*REN* is hypoproliferative anemia, which is probably secondary to low erythropoietin levels. This type of anemia disappears in the adolescence, which could be explained with increased production of sex steroids resulting in increased erythropoietin production (18, 25, 26).

Regarding the treatment of patients with ADTKD-*REN*, alongside the treatment of hyperuricemia and CKD, hypoproliferative anemia is treated with erythropoietin, hypotension and hyperkalemia can be treated with fludrocortisone (18, 25). Moreover, the potential role of BRD4780 can be taken into account, due to the fact that prorenin is accumulated in the kidney, similar to ADTKD-MUC1 (22).

### 1.5. ADTKD-*HNF1B*

Autosomal dominant tubulointerstitial kidney disease is also related to *HNF1B*, a gene that is located on chromosome 17q12 and is expressed in multiple tissues with an important role in early embryonic development, which could explain the complex presentations of the *HNF1B*-related disorders. They can affect many organs: the kidney and genitourinary tract, pancreas, and the liver. The loss of *HNF1B* gene function is one of the features of the 17q12 deletion syndrome. Studies suggest that a loss of one copy of the *HNF1B* gene in each cell causes the kidney and urinary tract abnormalities, as well as abnormalities of the pancreas that underlie diabetes (27–29). It was found that loss of *HNF1B* induces epithelial-mesenchymal transition and leads to kidney fibrosis (30). The loss of one copy of *LHX1*, also located on chromosome 17q12, is the likely cause of intellectual disability, behavioral and psychiatric conditions (27–31). Thus, ADTK-*HNF1B*, unlike other ADTKD forms presenting with CKD and gout, has a complex, syndromic presentation involving multiple organic systems. The MAGIC LUCID is helpful mnemonic which summarizes the clinical features of ADTKD-*HNF1B*, both renal and extrarenal: M = Hypomagnesemia; A = Autosomal dominant; G = Genital tract abnormalities, including bicornuate uterus, absent uterus, vaginal hypoplasia; I = Incomplete penetrance; C = Cysts of the kidney and other structural abnormalities, including multicystic kidneys, fetal bilateral hyperechogenic kidneys, kidney agenesis, and hypoplastic kidneys; L = Liver test abnormalities; U = Uric acid elevation; C = Chronic kidney disease; I = Inherited; D = Diabetes and pancreatic anomalies (32).

### 1.6. ADTKD-*SEC61A1*

The autosomal dominant tubulointerstitial kidney disease-*SEC61A1* is a disease associated with pathogenic variants, i.e., missense mutations in *SEC61A1,* a gene responsible for the encoding of the alpha subunit of the endoplasmatic reticular membrane translocon SEC61. The function of the SEC61 complex is crucial for the kidney development (10). The pathogenic variants cause SEC61-channelopathy due to the change in selectivity and permeability of the translocon channel. Moreover, the changed SEC61*α* accumulates in the endoplasmatic reticulum, leading to alterations in post-translational modifications and folding of various secretory and transmembrane proteins (uromodulin, mucin-1, renin) (10, 33). These alterations are causing endoplasmatic reticulum dysfunction and apoptosis, which leads to interstitial fibrosis (10). No specific treatment is available for ADTKD-*SEC61A1*, but recent data show that sodium phenylbutyrate (a small molecule approved for treatment of urea cycle disorders) may reverse the impairment of renin transport in ADTKD-*SEC61A1* (34).

### 1.7. ADTKD-*DNAJB11*

ADTKD-DNAJB11 is a very rare form of ADTKD with an atypical clinical presentation. Recently, five different heterozygous pathogenic variants in the *DNAJB11* gene were identified in seven families, consistent with autosomal dominant inheritance. The remarkable feature of ADTKD—*DNAJB11* is that the renal phenotype and clinical presentation markedly overlap with autosomal dominant polycystic kidney disease with small bilateral renal cysts, slightly enlarged kidneys, liver cysts, and slowly progressive renal failure. ESRD develops later than in other ADTKD forms, most commonly in the sixth or seventh decade (35). Thus, the diagnosis can only be made by performing genetic analysis and confirming the presence of a pathogenic variant in the *DNAJB11* gene (35). The *DNAJB11* gene produces a cofactor of GRP78/BiP, a major endoplasmatic reticulum protein, that has multiple functions in maintaining endoplasmatic reticulum homeostasis. Interestingly, histologic analysis of renal biopsy samples from affected patients also showed intracellular depositions of uromodulin and mucin-1 (35). There is no specific therapy available for ADTKD-*DNAJB11* (6).

## 2. Case reports

### 2.1. Case report—ADTKD-*MUC1*

**Figure 1:**
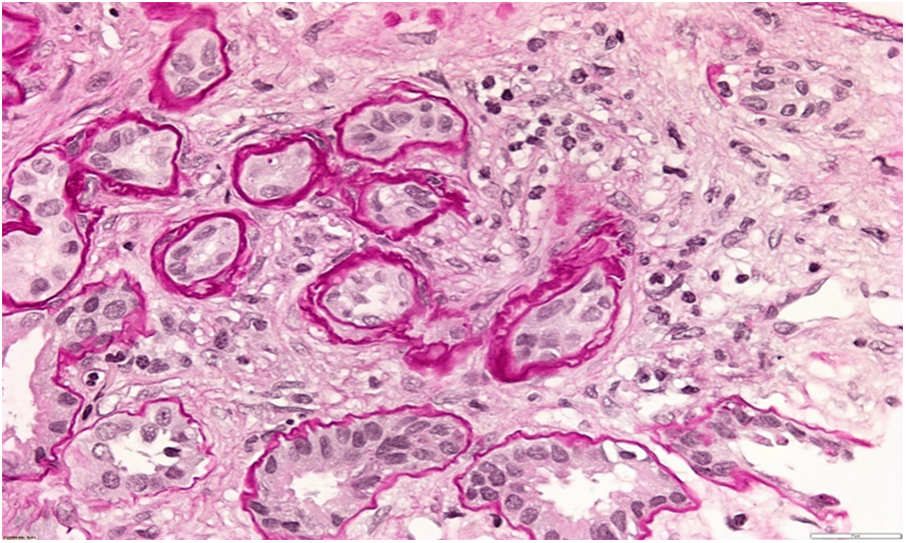
Kidney biopsy showing small foci of fibrosis in tubulointerstitium and stratified tubular basal membranes.

**Figure 2:**
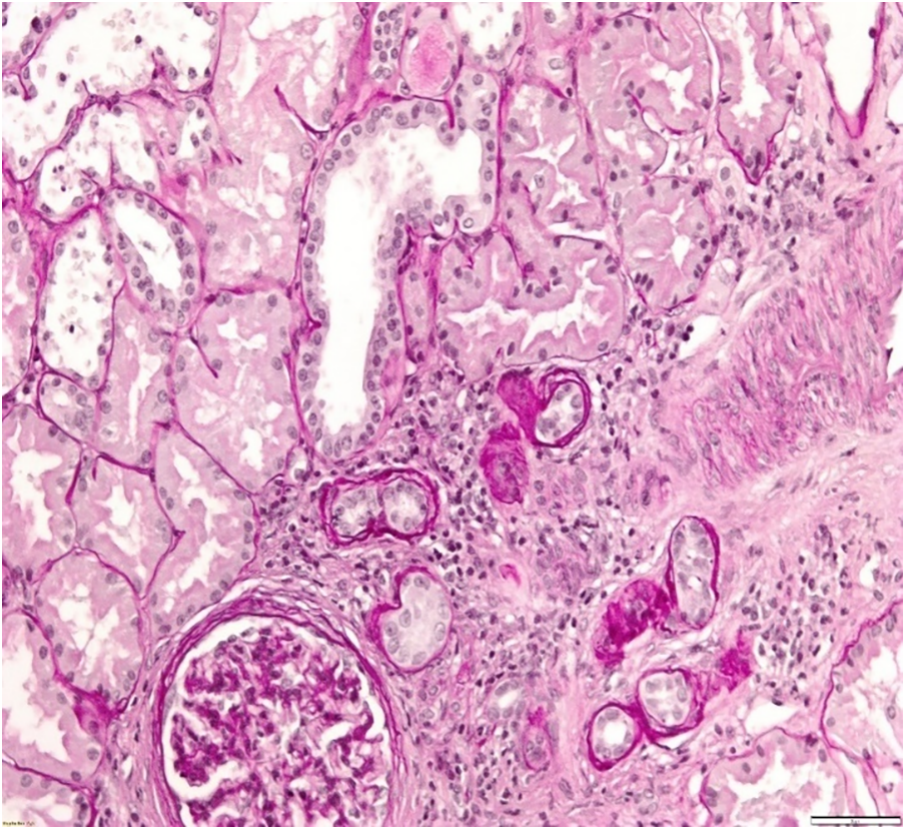
Kidney biopsy showing small foci of fibrosis in tubulointerstitium.

A 34-year-old patient was admitted to our department due to kidney injury. His serum creatinine levels were elevated (140–150 μmol/L, reference range 60–104 μmol/L, GFR 50 ml/min/1.73 m2). He had bland urinary sediment and no significant proteinuria (0.1 g in daily urine). Despite his slightly reduced GFR, he had hypoproliferative anemia with a hemoglobin level of 116 g/L. His blood pressure was slightly elevated, up to 145/95 mmHg. The kidneys appeared normal on the ultrasound exam. He had no history of other diseases, and he was taking no medication. His father and aunt also had chronic kidney disease. They both developed end-stage renal disease in adulthood (at 36 and 50 years of age, respectively), but the exact cause of ESRD was not established. We performed a kidney biopsy, which showed nonspecific chronic changes in the glomeruli and tubulointerstitium. Small foci of fibrosis in the tubulointerstium were observed, with stratified tubular basal membranes (Figures 1, 2). One small segment of glomerular sclerosis was found. The immunofluorescence finding was nonspecific, and electron microscopy showed no significant changes other than focal loss of podocyte foot processes, thus mimicking focal segmental glomerulosclerosis (FSGS). Considering positive family history, clinical data, and histopathological findings in the kidney, genetic analysis was performed in order to confirm ADTKD. DNA was extracted from an EDTA peripheral blood sample. A molecular genetic analysis of the genes *UMOD, HNF1 B, REN,* and *PAX2*, as well as parts of the *MUC1* gene, was without pathological findings. Targeted polymerase chain reaction (PCR) amplification of the VNTR-region between exons 2 and 3 of the *MUC1* gene was performed. Subsequently, amplicons were digested with Mwol and a proper mini-sequencing approach (SnaPshot Multiplex Kit, Life Technologies) was used to specifically prove the presence of the ADTKD-causing cytosine duplication c.428dupC in the *MUC1* gene (OMIM 158340; RefSeq NM_002456.5) (36). The generated fragments were separated with an automatic sequencing system (Applied Biosystems) and analyzed using the GeneMapper 4.1 software (37, 38). To determine the pathogenicity of the variant, the results were compared across public databases: OMIM (Online Mendelian Inheritance in Man), Database of Single Nucleotide Polymorphisms (dbSNP), US National Library of Medicine (Pubmed), Exome Variant Server (EVS), Exome Aggregation Consortium Browser (ExAC), Genome Aggregation Database Browser (gnomAD), Human Gene Mutation Database (HGMD), and Leiden Open Variation Database (LOVD) (36, 39–45). The detected sequence variant c.428dupC within the MUC1 VNTR (variable number of tandem repeats) leads to a shift in the reading frame, causing an early stop codon shortly beyond the VNTR domain, resulting in a protein lacking the transmembrane region and the cytoplasmatic domain, and is the prototypic pathogenic mutation in ADTKD-MUC1 (class 5-variant; HGMD-ID CP132082; Pubmed PMIDs 23396133, 24670410). The classification of sequence variants was done according to Wallis et al. (46). The finding of a pathogenic variant in MUC-1 confirmed the ADTKD-MUC1 diagnosis in our patient. The patient was treated with allopurinol, calcium channel blocker and an ACE inhibitor. In a six-year follow-up period, his kidney function was stable in stage G3. The clinical data are summarized in Table 2.

**Table 2:** Clinical course of ADTKD-*MUC1* patient.

Year	2016	2017	2018	2020	2022
Serum creatinine levels (*μ*mol/L, RR 70–104)	136	153–135	143	160	168
eGFR (ml/min/1.73 m2, RR > 60)	58	50–58	53	46	43
Serum urate levels (μmol/l, RR 182–403)	391	429	411	454	386
Serum potassium levels (mmol/L, RR 3.9–5.1)	4.3	4.4	4.6	4.4	4.1
Proteinuria (g/24 h urine, RR <0,25)	0.16	0.27	0.11	0.07	0.07
Haemoglobin (g/L, RR 138–175)	135	132	124	126	120
Office blood pressure (mmHg)	130/80	140/100	120/80	120/80	120/80
Therapy	lacidipin 4 mg + allopurinol 100 mg	lacidipin 4 mg + allopurinol 100 mg	lacidipin 4mg + allopurinol 100 mg	perindopril 5 mg + allopurinol 100 mg	perindopril 5 mg + allopurinol 100 mg
		Kidney biopsy was performed revealing nonspecific chronic changes in the glomeruli and tubulointerstitium, stratified tubular basal membranes, focal loss of podocyte foot processes on electron microscopy	Genetic analysis confirmed ADTKD-*MUC 1*		

RR, reference range; eGFR, estimated glomerular filtration rate; ADTKD, autosomal dominant tubulointerstitial kidney disease.

### 2.2. Case report—ADTKD-*HNF1B*

A 17-year-old male patient was referred to genetic counseling due to dysmorphic facial features, intellectual disability, and epilepsy. He had significant delays in motor and speech development and had been treated with valproic acid due to epilepsy since childhood. On physical examination, he had a height of 177 cm (50th centile), a weight of 69 kg (46th centile), a head circumference of 56 cm (25th centile), a long and narrow face appearance, a protruding chin, and prominent ears. His intellectual efficiency was between moderate and severe. Family history was negative for mental or kidney diseases. Additional workup was done, revealing hypomagnesemia (Mg 0.45 mmol/L, reference range 0.74–0.97 mmol/L). He had slightly elevated serum creatinine levels (92 μmol/L, reference range 55–90 μmol/L) with bland urinary sediment and no proteinuria—0.04 g in daily urine (Table 3). On ultrasound, the kidneys were of normal size but with impaired corticomedullar differentiation, hyperechoic parenchyma, and dilatated pyelons. The genomic DNA of the proband and, subsequently, that of his parents and sister was extracted from an EDTA peripheral blood sample. Array comparative genomic hybridization was performed using the SurePrint G3 Human 8 × 60 k Agilent oligo-microarray chip (Agilent Technologies, Santa Clara, CA, USA). Normal human male DNA (Agilent Technologies) was used as a reference. DNA labeling, slide hybridization, and washing were performed following the standard protocol provided by Agilent (47). A microarray slide was scanned in the Agilent Microarray Scanner System, and image data were extracted using the Agilent Feature Extraction software (v12.0.31). Agilent Cytogenomics software (v5.1.1.15) was used for the analysis of the results to determine copy number variations (CNVs). Genomic positions refer to the UCSC Genome Browser, February 2009, and the NCBI Build 37 reference sequence (GRCh37/hg19) (48).

**Table 3:** Clinical course of ADTKD-*HNF1B* patient.

Month/year	February 2018	April 2018	March 2019
Physical examination	Height: 177 cm (50th centile)		
	Weight: 69 kg (46th centile)		
	Head circumference: 56 cm (25th centile)		
	Long and narrow face protruding chin prominent ears		
	Intellectual efficiency was between moderate and severe		
Haemoglobin (g/L, RR 129–166)	143		
Serum urate levels (μmol/l, RR 182–403)	571		550
Serum creatinine levels (μmol/l, RR 40–72)	92		88
Serum fasting glucose levels (mmol/L, RR	6.4	7.2	7.4
Serum magnesium levels (mmol/L, RR 0.74–0.97)	0.47	0.46	0.55
Serum potassium levels (mmol/L, RR 3.9–5.1)	4.2	3.8	
Proteinuria (g/24 h urine, RR <0,25)		0.04	
Therapy	diazepam 5 mg magnesium citrate 234 mg sodium valproate 500 mg		
	Genetic analysis confirmed ADTKD -*HNF1B*		

RR, reference range; ADTKD, autosomal dominant tubulointerstitial kidney disease.

**Figure 3:**
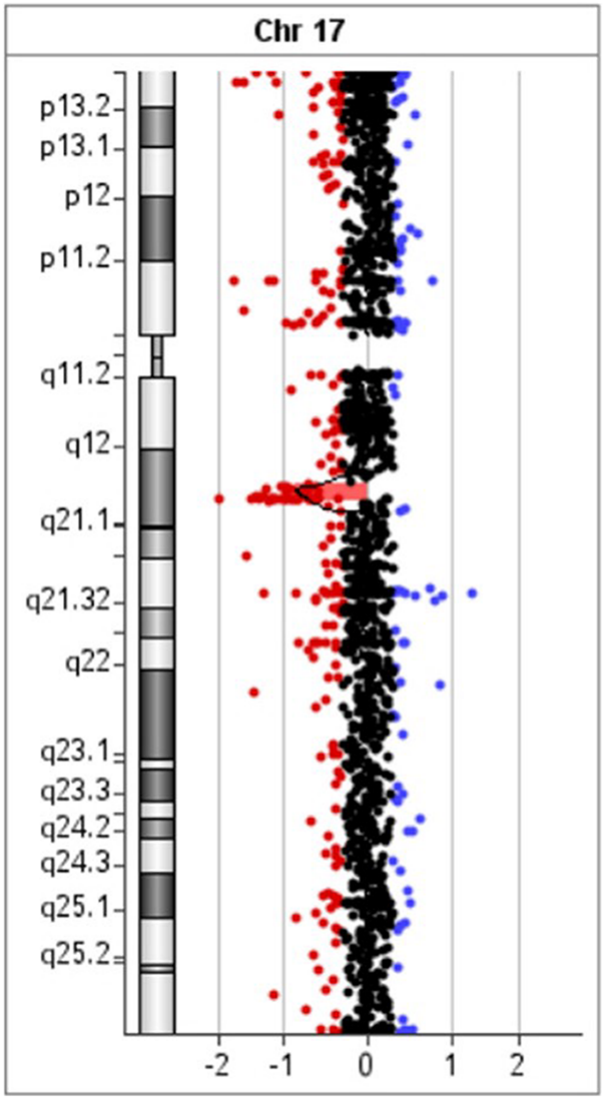
Array comparative genomic hybridization of the proband showed a 1.39 Mb heterozygous interstitial deletion at 17q12 arr[GRCh37] 17q12(34856055_36248918)x1.

**Figure 4:**
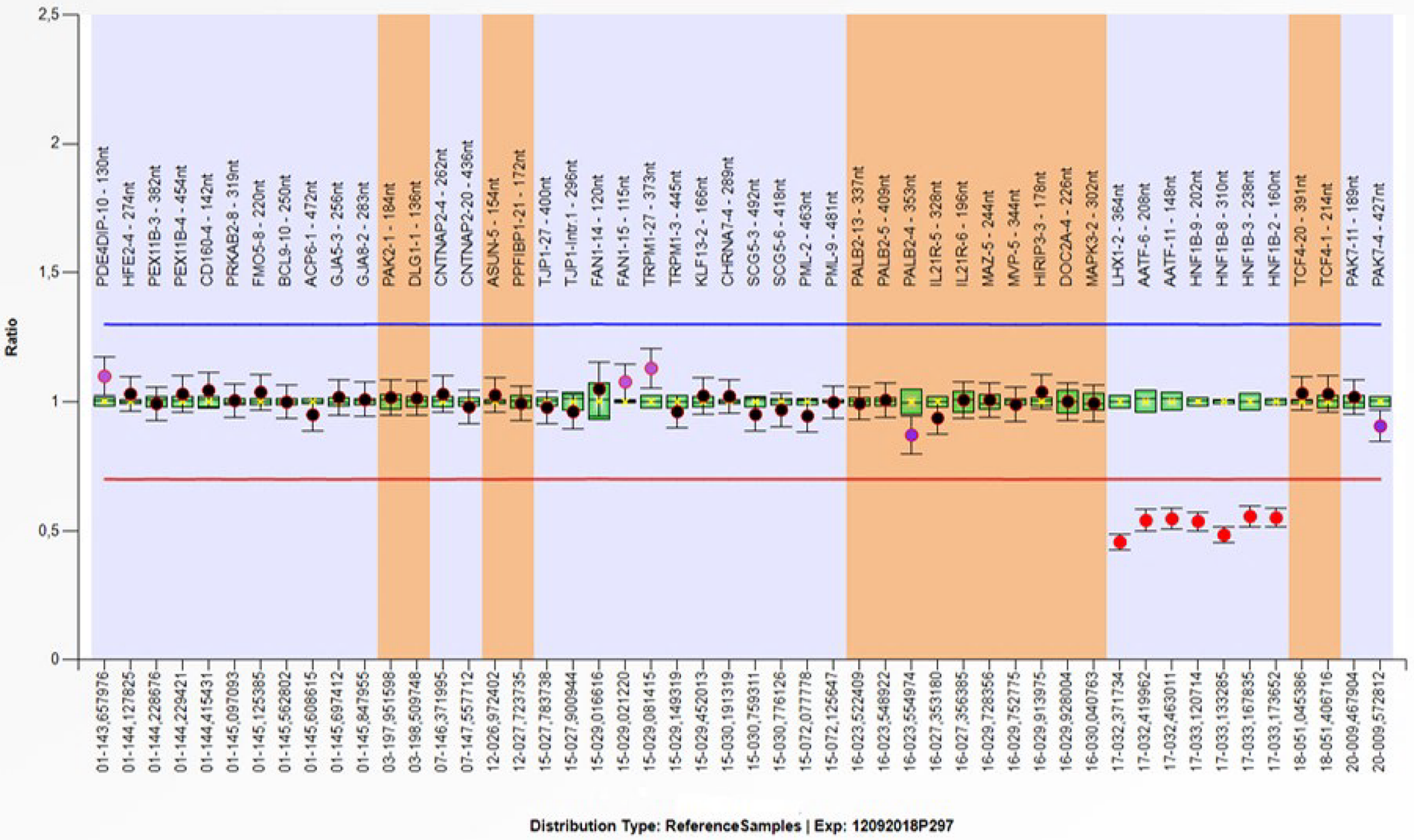
MLPA (Multiplex ligation probe amplification) SALSA MLPA probemix P297-B2 microdeletion syndromes -2 (MRC-holland): rsa 17q12(LHX1,AATF,HNF1B)x1 mat.

Array-CGH data were confirmed by using MLPA (Multiplex Ligation Probe Amplification) with the SALSA MLPA kit P297-B2 Microdeletion Syndromes-2 (49). The MLPA reaction was performed according to the manufacturer's standard protocol and reagents (MRC Holland, Amsterdam, the Netherlands) (50). Amplified products were recognized and quantified by capillary electrophoresis on an ABI 3,130 Genetic Analyzer (Applied Biosystems, Foster City, CA, USA). For MLPA analysis, raw data were exported into the software Coffalyser (MRC- Holland). To determine the pathogenicity of CNVs, the microarray results were compared across public databases: DECIPHER (Database of Chromosomal Imbalance and Phenotype in Humans Using Ensemble Resources), OMIM (Online Mendelian Inheritance in Man), ClinGen (Clinical Genome Resource and ISCA), PubMed, and population database DGV (Database of Genomic Variants) (36, 40, 51–53). Array-CGH analysis revealed a male molecular karyotype with a 1,392 kb heterozygous interstitial deletion on the long arm of chromosome 17 involving region q12 [hg 19 coordinates chr17: 34,856,055-36,248,918] according to the UCSC Genome Browser (GRCh37/hg19 assembly) (Figure 3). Copy number variations (CNVs) reported in the Database of Genomic Variants were excluded from further analysis. The deleted region 17q12 contains 19 protein-coding genes, three of which are reported in the OMIM Morbid Map: Hepatocyte nuclear factor-1-beta *HNF1B* (OMIM ID 189907), Acetyl-CoA carboxylase alpha ACACA (OMIM ID 200350), and phosphatidylinositol glycan anchor biosynthesis class W protein PIGW (OMIM ID 610275). The deletion was confirmed by MLPA (Multiplex Ligation Probe Amplification) using SALSA MLPA probemix P297-B2 Microdeletion Syndromes-2 (MRC-Holland). MLPA analysis revealed the loss of *LHX1, AATF*, and *HNF1B* genes in the patient, his mother, and his sister (Figure 4), and a normal copy number for all genes of this probemix in the father (54). The patient was treated with magnesium suspension therapy, diazepam, and valproate. During the follow-up period, he didn't develop any new health issues, and his kidney function remained stable. In the meantime, his mother developed MODY5 diabetes. She had discreetly reduced renal function with mild hydronephrosis of the right kidney and multiple kidney cysts. Medical records of the patient's sister were not available for this report.

## 3. Discussion and conclusion

ADTKD includes different genetic forms (ADTKD-*UMOD*, ADTKD-*MUC1*, ADTKD-*REN*, ADTKD-*HNF1B*, ADTKD-*SEC61A1*, and ADTKD-*DNAJB11*) of slowly progressive kidney disease. The positive family history compatible with autosomal dominant inheritance and CKD with the clinical characteristics of tubulointerstitial kidney disease should raise suspicion of ADTKD (13). If there is no family history of CKD, clinical suspicion should be raised based on clinical characteristics, including early onset of hyperuricemia or gout, and compatible histology on a kidney biopsy (if available). In the case of *HNF1B*-related disease, there should be extrarenal manifestations of the disease. The diagnosis is based on a positive family history and compatible kidney histology in at least one affected family member, with a demonstration of a pathogenic variant in one of the aforementioned genes in an affected individual or at least one family member (13). The role of kidney biopsy in diagnosing ADTKD is controversial since there is no pathognomonic finding and histological changes can mimic focal segmental glomerulosclerosis (FSGS), much like in our ADTKD-*MUC1* patient. The clinical findings (reduced GFR of 50 ml/min/1.73 m2, bland urinary sediment with no significant proteinuria) were not consistent with the diagnosis of FSGS, which made us consider genetic analysis in order to establish the correct diagnosis. Although hypo proliferative anemia is more common in ADTKD-*REN*, our patient had low values of hemoglobin at diagnosis (116 g/L). It is reported that ADTKD-*MUC1* has a more aggressive clinical course in comparison to ADTKD-*UMOD*, with an earlier onset of ESDR (mean age 45 years) (17).

Our ATDKD-*MUC1* patient, however, has a stable kidney function in stage G3 at age 40.

Regarding our patient with ADTKD-*HNF1B*, he had classic clinical features of the disease (CKD without significant proteinuria, bland urinary sediment, hypomagnesemia, neurologic disturbances, and kidney structural abnormalities). Family history seemed to be negative; however, the genetic analysis revealed the diagnosis and AD inheritance consistent with ADTKD-*HNF1B* disease.

Deletions within the 17q12 region, which contains the *HNF1B, ACACA*, and *LHX1* genes, are consistent with a diagnosis of recurrent 17q12 deletion syndrome. We have been searching in the Decipher and other databases for relatively similar-sized deletions at 17q12 and have identified over 70 cases with certain pathogenicity, pointing to the mostly characteristic phenotype of this syndrome. In this way, the recurrent nature of deletions and duplications is thought to be mediated by flanking segmental duplications or low-copy repeats (LCRs) that increase the likelihood of copy number variation through nonallelic homologous recombination (NAHR). The presence of region-specific low-copy repeats (LCRs) is commonly caused by NAHR between paralogous genomic segments (55)..

*HNF1B* is a transcription factor-2 (TCF2), whose expression was detected in the kidney, liver, bile ducts, thymus, genital tract, pancreas, lung, and gut (56). Haploinsufficiency of the *HNF1B* gene is responsible for most of the features associated with the 17q12 microdeletion syndrome. *LHX1* plays a role in the differentiation of neural cells and the transcriptional control of axonal guidance and has been associated with epilepsy, autism, and intellectual disability (57). The *ACACA* gene encodes acetyl-CoA carboxylase, an important enzyme in fatty acid synthesis. The recurrent 17q12 deletion is a pathogenic variant with incomplete penetrance and variable expressivity. 30 As reported, the estimation risk for DD/ID of penetrance was about 34.4% for the 17q12 deletion, including the *HNF1B* gene (58). There is no specific treatment for any form of ADTKD. The data about the possible beneficial effect of treatment with angiotensin-converting enzyme inhibitors or angiotensin receptor blockers on CKD progression in ADTKD patients is lacking. Patients with *UMOD*-related disease who develop gout will probably have relapses and therefore should be treated with allopurinol or febuxostat (13, 59). It is not known if a strict diet with low purine content is beneficial in patients with hyperuricemia with *UMOD* mutations. Diuretics should be prescribed cautiously in all patients with ADTKD, as they may worsen hyperuricemia and volume depletion (60). Increased fluid intake is recommended to compensate for possible urine concentration dysfunction. A low-sodium diet is not recommended for ADTKD-*UMOD* and ADTKD*-REN* patients, as decreased salt intake may worsen hyperuricemia and volume depletion. 13 Non-steroidal anti-inflammatory drugs should be avoided in all patients with ADTKD, especially in patients with *REN* mutations who are very susceptible to acute deterioration of renal function. In patients with ADTKD-*REN*, erythropoietin and fludrocortisone may be used for the treatment of anemia and symptomatic hypotension, respectively (13, 61). Fludrocortisone should not be prescribed in patients with deteriorating renal function, arterial hypertension, hypokalemia, and edema (61). All ADTKD patients are good candidates for kidney transplantation since there is no disease recurrence in the graph (6).

In summary, our two cases emphasize the importance of considering ADTKD within the wide range of differential diagnoses when examining a patient presenting with CKD, even in the absence of family history and other suggestive symptoms and signs. We believe that further reports of ADTKD cases might aid in improving the recognition and care of this intriguing disease group.

## 4. Patient perspective

The ADTKD—*MUC1* patient has been worried about his health since he was young, due to an unknown kidney disease present in the family. Although the diagnosis was established, he is still troubled by the fact that his future remains uncertain.

The mother of the ADTKD-*HNF1B* patient consulted a pediatrician due to the suspicion of a hereditary disease in her son, although the family history was not clearly positive. The diagnostic work-up confirmed her suspicions. Although her son's condition is now stable, he needs constant care and attention.

## Data Availability

The datasets presented in this study can be found in online repositories. The names of the repository/repositories and accession number(s) can be found in the article/Supplementary Material.
